# Why research ethics should add retrospective review

**DOI:** 10.1186/s12910-019-0399-1

**Published:** 2019-10-10

**Authors:** Angus Dawson, Sapfo Lignou, Chesmal Siriwardhana, Dónal P. O’Mathúna

**Affiliations:** 1 University of Sydney, Sydney, Australia; 2 University of Oxford, Oxford, UK; 3 Anglia Ruskin University, Chelmsford, UK; 4 Dublin City University, Dublin, Ireland; 5 The Ohio State University, Columbus, USA

**Keywords:** Research ethics, Integrity, Virtues, Prospective review, Retrospective review

## Abstract

Research ethics is an integral part of research, especially that involving human subjects. However, concerns have been expressed that research ethics has come to be seen as a procedural concern focused on a few well-established ethical issues that researchers need to address to obtain ethical approval to begin their research. While such prospective review of research is important, we argue that it is not sufficient to address all aspects of research ethics. We propose retrospective review as an important complement to prospective review. We offer two arguments to support our claim that prospective review is insufficient. First, as currently practiced, research ethics has become for some a ‘tick box’ exercise to get over the ‘hurdle’ of ethics approval. This fails to capture much of what is important in ethics and does not promote careful reflection on the ethical issues involved. Second, the current approach tends to be rules-based and we argue that research ethics should go beyond this to develop people’s capacity to be sensitive to the relevant moral features of their research, their ethical decision-making skills and their integrity. Retrospective review of a project’s ethical issues, and how they were addressed, could help to achieve those aims better. We believe that a broad range of stakeholders should be involved in such retrospective review, including representatives of ethics committees, participating communities and those involved in the research. All stakeholders could then learn from others’ perspectives and experiences. An open and transparent assessment of research could help to promote trust and understanding between stakeholders, as well as identifying areas of agreement and disagreement and how these can be built upon or addressed. Retrospective review also has the potential to promote critical reflection on ethics and help to develop ethical sensitivity and integrity within the research team. Demonstrating this would take empirical evidence and we suggest that any such initiatives should be accompanied by research into their effectiveness. Our article concludes with a discussion of some possible objections to our proposal, and an invitation to further debate and discussion.

## Background

We argue that the current system of research ethics review does not achieve its central objective of ensuring that research is conducting in the most ethical way possible. One reason for the limitations of current practice is that the contemporary research ethics review model is not capable of addressing all aspects of research ethics. We hold that this is, in part, due to ethics review being almost solely *prospective* in nature. Research ethics committees (RECs), also called institutional review boards (IRBs), spend the majority of their time focused on reviewing studies prospectively using the standard paperwork required by the particular national or institutional system. Occasionally, a REC may require researchers to attend a meeting, largely to answer questions arising from the submitted materials. The REC deliberates based on the submitted information, using its collective experience and prior knowledge about other related research to make a judgment about potential risks and benefits of the study under review. Such review processes are important, but they are insufficient, because all this occurs after the project has been designed and prior to the commencement of the research.

Ethical issues arise at many other points in research. Sponsors and funders influence the choice of what gets researched in the first place, while a lack of engagement with participant communities during the research design can lead to ethical challenges during implementation. Ethical issues arise during projects, especially with research in humanitarian settings, which is our particular interest, due to the instability and insecurity in such contexts. A regulatory and pre-approval approach to research ethics, focused on well-known ethical principles and issues, like informed consent or confidentiality, cannot address everything that is crucial in research ethics. What is missing “is how to positively encourage ethical conduct. Developing an understanding of what to do is always a more challenging prospect than issuing edicts about what is not right” [1]. The contemporary review process lacks ways to develop the reflexivity and ethical sensitivity necessary to promote decision-making skills during ethical challenges and dilemmas [2]. Research integrity and using virtue ethics in research training underlie some initiatives seeking to address short-comings in the current system [3].

We suggest that the current prospective system of ethics approval tends to focus on a small number of well-trodden ethical issues which can leave other important ethical issues unexamined. On this standard approach, research ethics review happens in a moment in time, occurring before the research itself is conducted. We argue that such an approach is not sufficient to address all of the important ethical issues that arise during research. One possible alternative, that has been discussed somewhat in the literature, is on-going or continuing ethics monitoring during the course of the research. We discuss some difficulties with this approach and suggest an alternative. We set out the option of conducting retrospective review of research in addition to prospective review, in at least some cases, and argue that this would be beneficial in regards to achieving the overall aims of health research ethics. We respond to questions that our proposal may raise: How might this be done? What are the potential benefits? Who might benefit? What are the potential difficulties with such an approach? We begin by providing some reasons to think that the present solely prospective system is inadequate.

## Why prospective research ethics review is not enough

We offer two arguments for why the present system of prospective research ethics review is inadequate. The first argument is that it encourages a procedural and legalistic approach to research ethics and fails to capture what is important to ethics. Of course, this objection is a long-standing one in research ethics [4–6]. However, we suggest that prospective review encourages the idea that research ethics is a hurdle or a hoop, something to get over or through. By focusing on ethical issues solely in advance of conducting research the idea is encouraged, even if only implicitly, that research ethics is a tick-box exercise; if the forms are filled in correctly, then ethics is ‘done’. Discussion of research ethics is now ubiquitous in the research community, but “researchers complain that institutional review boards have lost sight of their original purpose of protecting human subjects, focusing instead on bureaucratic minutiae” [7].

Our concern about how research ethics becomes more procedural has two variants, both of which are relevant to our critique of prospective accounts of research ethics. First, the external observer might be forgiven for thinking that the focus is upon process, in the sense that research ethics is about the establishment and refinement of a set of routines for the review of research. A committee is established with formal criteria for membership (e.g. clinicians, researchers, pharmacists, social scientists, ethicists, lay members, etc.) and a set of standard operating procedures (e.g. required application forms, fixed questions, deadlines, etc.). This idea can also encourage the members of RECs to focus on minutiae and the textual, rather than the ethical issues as such. Some of us have experienced being on RECs where the focus seems to be more on the misuse of apostrophes rather than the ethical challenges and problems in the research itself.

The second variant of our objection is how a procedural approach to research ethics review tends to favour a formalisation of the ethical issues, in the sense that research ethics guidelines are increasingly framed as sets of rules (where such rules are often interpreted as being of absolute form – that is, without exception). Again, our experience as members of RECs suggests that at least some members cling to the ‘rules’ (as they see them) and resist thinking about why it may (or may not) be appropriate to follow them in any particular case. The basis for this commitment to both forms of proceduralism is, presumably, the thought that any research protocol ‘passed’ by the correctly constituted REC that applies the right guidelines in the right way will, in some sense, be guaranteed to be ethical. Of course, this does not follow. We suggest that this legalistic approach to research ethics is problematic, and that it is time to return *ethics* to the heart of research ethics review. This alternative view sees ethics as being very different, not so much an application of rules, which primarily seem to provide legal protection for the institution [4, 8], but rather being about developing people’s capacity to be sensitive to the relevant moral features of the particular research context, and taking responsibility for the moral judgments that arise from responding to the dynamic nature of each individual piece of research. No review process can address or respond to all aspects of research ethics. Regulation and ethical approval have an important role, but this must be combined with other commitments, such as to ethics education, skill development and character formation.

The second argument for holding prospective review alone to be problematic is that such an approach must necessarily be insufficiently sensitive to the right features relevant to making moral judgments. The prospective questions asked as part of the research ethics paperwork tell us something about relevant ethical issues, but by their nature they cannot capture the complexity of what is relevant in each individual case. A retrospective ethics review provides a better opportunity to critically reflect upon all of the relevant considerations that arose in the context of a particular project. To give just one example for now, in prospective review researchers are asked to state what they believe will be the likely benefits and possible harms that might result from the research. Such calculations are important, but by definition these can be, at best, only probabilistic predictions. During the conduct of a study researchers may identify new risks or benefits, or develop innovative ways to reduce risks, or come across new information that changes how risks and benefits may be balanced. Once a project is complete, reflection upon what happened would allow discussion about the harms and benefits that *actually* arose and how those compared with what was predicted. We acknowledge that such reflection does occur during and after some research, but is largely informal and not disseminated in ways that allow others to learn from such experiences. This is particularly important when conducting research in a humanitarian, emergency or conflict situation where ethical lessons learned from urgent actions undertaken during the research would be useful [9]. Such retrospective review could also be important in non-crisis situations, where, while the context may have less intensity and complexity, it is still important to learn lessons from completed research.

Despite its flaws, the current system of prospective review does make an important contribution to bringing about research that can be seen to be more ethical. For example, it ensures that people external to the research examine at least some of the ethical issues before the research begins and this, arguably, provides an incentive for researchers to produce protocols that take ethical issues more seriously than they otherwise might [10]. As we explain later, prospective ethics review does achieve some of the relevant goals of research ethics, and therefore we should not be taken to be arguing that the current system be abolished, but, rather, that it should be complemented by retrospective ethics review. In addition, we are not suggesting that every research project requires an extensive retrospective review process. Such a requirement could become excessively burdensome in ways that prospective review can be. Low-risk research using standardised methods may not require as extensive a review as high-risk, innovative and challenging projects. In humanitarian research, the settings are particularly demanding, participants are often vulnerable in many ways, protocols may have to adapt to changing circumstances, and therefore retrospective review is particularly important [9]. At the same time, all researchers should reflect on the lessons that could or should have been learned, even with low-risk research.

## Why on-going ethical monitoring is not sufficient

Many alternative models of ethical governance have been suggested to address the limitations of prospective review. Some literature has proposed that a process of on-going or continuing ethical monitoring should be implemented for research that has been previously approved [4, 11–14]. Such procedures could take many forms, such as the review of progress reports, annual re-review and re-approval, monitoring of data integrity, announced or unannounced site visits, monitoring of the quality of informed consent processes, etc. Another possible model would be to expand the role of data and safety monitoring boards (DSMBs) to include continuing review of ethical considerations. At the moment, any such existing on-going monitoring bodies have very specific and restricted roles. The focus of such bodies could, of course, be expanded and this might be particularly useful where research participants are exposed to more than minimal risk or when vulnerable populations are involved [15–18] or if complaints are made to the REC or IRB.

However, despite the possible expansion of on-going ethical monitoring, and a possible role in helping to address some of the day-to-day problems that might arise in research, we do not believe that it alone provides sufficient response to the problems in the current prospective system as outlined in the previous section. On-going monitoring, where adopted, often accepts minimal reporting such as annual or end-of-study reports [14]. It can result in the same kinds of procedural problems that arise in prospective review. The focus is very likely to be on completing reports rather than critical reflection, engaged discussion, and an increased sensitivity towards all aspects of relevance to ethical judgment. Of course, retrospective review could also be approached in the same way and similarly fail to achieve its overall aim. Steps to avoid this will be considered below. In addition, the literature suggests a number of reasons for scepticism in seeing on-going monitoring as a solution, for practical reasons. For example, RECs and DSMBs do not have the expertise and will often lack the capacity to monitor on-going studies, especially if RECs and DSMBs are in low-income countries where resources are limited and infrastructure weak [19–21]. Whilst reporting may be required for adverse events and a very light touch report may be required if the research continues beyond a set period (often 12 months), only a small percentage of studies are monitored post-approval [15, 22–24]. We know of no actual REC that requires systematic consideration of ethical issues once the initial ethics approval is obtained from prospective research ethics review.

## The aims of retrospective ethics review

The reader will no doubt already have a number of questions about what exactly we are proposing. We are not suggesting that retrospective review should simply replace prospective review as the primary means of assessing research protocols. We acknowledge that educational initiatives, training and mentoring that encourage reflection on ethics and character formation are required alongside any mechanism for research ethics review. In this paper we are seeking to examine the difference it might make to review the ethics of particular research projects after they have been conducted, as well as before. We leave it an open question, here, as to how exactly this ought to be done. Different approaches will suit different types of research and research settings, and it is very much not our aim to introduce yet another formalised procedure that must be followed. Whatever process is developed, it should seek to promote reflection and dialogue that leads to improved ethical practice and deeper concern for the well-being of participants. The exact method ought to follow from the specific nature and context of the project, and could include a research team meeting focused on the ethical issues that arose, a discussion amongst the members of a REC, a focus group with community members, or a survey of participants, etc. Every project will not require the same intensity in retrospective review, but this should be matched to the risk, innovation and complexity of the research. The important aspect is that open, honest reflection on ethical issues should be encouraged so that it contributes to a more accurate assessment of the actual complexities arising in research, including the actual risks and benefits. Discussions of this type may occur informally in some research groups, but they are rarely reported. We believe that without reports of these discussions, valuable lessons that were, or could have been, learned about the ethical issues arising during research are being lost, or become part of researcher folklore rather than contributing to more evidence-based research ethics.

It is important to see that retrospective review, as an additional feature of research ethics review, may have a number of possible aims as well as a number of possible benefits. We propose that the additional benefits should be considered for the widest possible number of stakeholders, something we analyse in detail in the next section. The kinds of aims that might be important for retrospective review could include: identifying new insights and knowledge about ethical issues from looking back at research already conducted, increased sensitivity of researchers to relevant ethical issues, learning lessons from adaptations made during the research to how ethical issues were addressed, contributing to the development of ethical standards and guidelines in research, etc. Depending on the aim or aims chosen, the type of study and the research population involved, different parties will be central to the relevant analysis and discussion. The list of who could be involved in retrospective review will include the researchers within each project themselves, teams of researchers working on parallel projects exploring common themes, members of research ethics committees, community members and/or their representatives in advocacy groups, participants, etc. Once it is decided who *might* be involved, we can then ask who *should* be involved. Deciding this goes to the core of how retrospective review may be a better means to achieving the overall aims of research ethics and improving the ethical judgments involved.

## The benefits that might follow from retrospective ethics review

We suggest that there are three main sets of potential benefits from conducting retrospective research ethics reviews. Firstly, the different stakeholders involved could benefit in various ways from improving the research ethics process; secondly, the moral character, integrity and sensitivity of these stakeholders could potentially improve; and thirdly, a greater degree of understanding of relevant ethical considerations could develop. We will consider each of these in turn, acknowledging that these are *possible* benefits. Retrospective research ethics review should be introduced along with mechanisms to assess its impact so that it contributes to evidence-based ethics review.

We consider first the benefits to each set of stakeholders in the research ethics process. For example, each principal investigator and research team may gain from reflecting retrospectively upon each study and discussing what went right and wrong, what could have been improved, and what can be learnt from that project and applied within the next project. This might be seen as similar to audit in clinical practice settings, where clinicians review cases to learn and improve future practice [18, 25]. Just as audit aims to improve patient care, the additional retrospective review we propose aims to improve how research is conducted. As mentioned above, retrospective review is likely to result in a more accurate assessment of harms and benefits (precisely because they can be judged retrospectively) and allow explicit consideration of any ethical issues that were missed during prospective review or allow reflection upon potential issues that were anticipated but did not actually emerge. Committees could learn from such reviews and so improve future prospective reviews, or identify situations which require closer oversight in future, similar research. Retrospective review, if conducted openly and honestly, could promote greater transparency and fairness in assessment of research and this may help identify common values, different priorities, and potential disagreements between stakeholders and help consider how these might be resolved. For example, ethical disagreements may be traced to misunderstandings between researchers and participant communities which could be addressed better in future research with similar populations. We see such a dynamic learning process as being particularly important and beneficial in a humanitarian research context, where the research participants may be disadvantaged in general or vulnerable in various different ways, where there is a greater potential for misunderstandings and complexity due to cross-cultural factors, an urgent need for action, and possibly on-going conflict. Rules and guidelines don’t apply as well when researchers are in such contexts and where time pressures may have impacted the prospective review process. Retrospective review could be particularly important to conduct with humanitarian research where ethics committees may have little expertise and experience with those types of research and their contexts [26].

The previous paragraph noted ways that members of one research team might benefit from retrospective review. In addition, a number of research teams could conduct joint reviews in which they share their experiences with each other and reflect upon them together. This could be particularly useful if they worked in a similar research area or geographical region, or used the same methodology. Such a ‘between the groups’ version of retrospective review could have many of the same potential benefits as ‘within the team’ review, but also some additional benefits. For example, each team could learn from the experience of how other teams address ethical issues, or learn about additional cultural factors in their participant communities that their team had not noticed previously. Dissemination of the learning from retrospective review could be made available openly, with due consideration made for confidentiality, so that it can be used for quality improvement and better protection of participants, researchers and other stakeholders.

Retrospective ethics review could also be opened up to those beyond the research teams. Members of RECs, participants, community members, or representatives of various stakeholders could be included. The various stakeholders could then contribute to reflections about the ethical issues that arose, whether perceived at the time or not. Integrating all stakeholders, particularly participants, in such reviews could bring to everyone’s attention how the ethical issues were perceived from different perspectives. Such an approach may allow all stakeholders to become better at making predictions about possible harms and benefits in future projects. For example, perhaps RECs systematically overestimate risks or researchers overestimate benefits. Future participants will benefit if everyone becomes aware of such trends and changes practice accordingly.

Such retrospective analysis might even be seen as a form of community engagement, if community members or their representatives can raise concerns and offer their views on the completed research and the ethical issues that arose. They could propose how things might be addressed differently in the future. In this way researchers, funders and ethics committees could incorporate due consideration of the moral concerns and priorities of the researched communities giving them a more meaningful voice in shaping future projects [27]. Finally, retrospective review may increase the likelihood of community-wide adoption of a new intervention, should it prove successful, strengthen the voice of the most disadvantaged researched communities, increase awareness of the social purpose of a health study or serve as a capacity-building method for local researchers in collaborative health research in developing settings.

The second general set of benefits may be to the different stakeholders in relation to the development of their characters, ethical sensitivity, and ability to detect and appreciate ethically relevant factors in research. The improvement here might be in terms of what we could characterise as the stakeholders’ virtues, perhaps most centrally a commitment to transparency, humility, and a willingness to change. For example, researchers might commit to transformation after talking freely and openly to their colleagues, their study participants and their communities. Through retrospective review researchers are not only likely to become more technically competent, but also develop in their individual and collective ability to make ethical judgments.

Third, such retrospective review might aid better understanding of, and promote the application of, relevant ethical values and principles in research ethics. As was suggested above, an alternative to thinking about ethics as a series of rules is to see ethical judgment as being about the sensitive response to ethically relevant, socially embedded features that together shape how we ought to act [28]. Retrospective review might well show how certain guidelines do not fit a particular case, do not apply in a humanitarian context (if our focus is on humanitarian health research), or have to be refined or developed (in at least some cases). Perhaps the insights from one area of ethics could come to be understood as being relevant in a new sphere. For example, perhaps ideas from public health ethics, such as a focus on common goods rather than individualistic values, result in a transformation of thinking in research ethics, through greater debate and reflection. Here it is the field of research ethics itself that stands to benefit.

## Responding to some possible objections

A number of possible objections can be raised to retrospective review. The force of each of these will differ depending upon the form which retrospective review takes and who is involved. Different types of research warrant different degrees of retrospective review depending on the complexity and innovativeness of the methods, the vulnerability of the participants, the setting of the project, and other factors. Each of these will generate different objections, and result in various responses. A comprehensive survey of all these lies beyond this paper, but we will consider three more substantive objections to our proposal here.

The first possible objection is that research ethics review is already too burdensome. We can easily imagine researchers rolling their eyes at the very idea of introducing a new ‘hurdle’ to be jumped. It is, of course, possible that retrospective review could become bureaucratic very quickly. It could be seen as one more report to be completed before being finished with ‘ethics.’ However, a lot would depend upon the proposed aim of any retrospective review procedures. If the focus is on developing the character of researchers and seeking greater sensitivity to ethically relevant factors, this should not add further unnecessary burdens to RECs or researchers or make research ethics any more bureaucratic. We are not proposing a formalised additional process like the current prospective review system. Indeed, our contention, as outlined above, is that it is precisely because current research ethics structures often miss what is essential to ethics, that we need something in addition to prospective review that can help to return research ethics to its original purposes: to help research and researchers become more ethical, and thereby respect and protect participants. Our proposal will achieve its aims only if developed within an approach to research ethics that returns it to its foundations in promoting respect for and protection of participants while promoting research of the highest rigour and quality. We propose that retrospective review is first conducted by researchers themselves, because they are (or should be) committed to improving the ethics of their own research. It might be that early enthusiasts establish good practice and provide different models demonstrating the potential benefits. This process may in turn be encouraged by research funders. RECs may also want to think about how a retrospective perspective might benefit their future decision-making.

The second possible objection is that retrospective review would not be necessary if adequate support and funding for conducting research ethics review were available. For example, given a choice, perhaps on-going ethical review by a REC would be even better than retrospective review. However, such continuing review would include retrospective review and, as already discussed, several issues make on-going review difficult to implement in practice. Retrospective review might be a compromise, with better prospects for successful implementation because it is easier, quicker and less costly.

The third objection might be that such reviews are bound to fail because researchers will be fearful about being transparent about problematic ethical situations. Perhaps researchers might, legitimately, dread opening themselves up to being sued by participants, being shunned by other researchers, being restricted from accessing future funding, having difficulty getting work published or having papers retracted. Retrospective review could uncover serious ethical problems or violations, although our proposal is not meant to introduce an ethics police force. The aim of retrospective review should be to learn from past experience. If researchers made ethical judgments in the midst of unexpected developments or even in a crisis, and sought advice from those available to assist, then the focus should remain on what can be learned, not on punitive measures. However, it must be acknowledged that some identified problems could lead to criticism or even negative consequences for researchers. At the same time, If problems are identified, these need to be addressed. If researchers must face consequences for serious ethical violations, then retrospective review will have served a role in ensuring these are noticed and receive a response. An unwillingness to conduct retrospective review because it may bring ethical violations to light should lead to questions about the ethics and integrity of such research. We argue that in contrast to additional levels of review or monitoring that would impede the timely conduct of a study, post-research ethics analysis and reflection could facilitate ethical research by building trust and by reducing inequalities of knowledge and power between researchers, ethics committees, funders and participants.

## Conclusions

We have proposed a number of potential benefits, to a range of relevant stakeholders, to conducting retrospective ethics review of research in addition to prospective review. We have not argued for any particular process or format for such review. The intention behind this paper is instead exploratory, to raise a series of questions to encourage debate. However, we do believe that current research ethics practice misses its target and has become lost in formulaic processes, and that considering retrospective review is one possible way to return ethics to the heart of research ethics review. Of course, our speculation about all of these potential benefits highlights the importance of empirically testing our proposal, and of the general need to develop evidence-based research ethics practice.

We are involved in empirical research to gain a better understanding of the experiences of researchers, research ethics committees, advocacy groups, etc. in meeting the ethical challenges that can arise in conducting research in humanitarian settings.^1^ This is a first step in developing a tool to facilitate post-research ethics analysis. We hope that such an evaluation tool will facilitate reflexive exploration of the ethical challenges that arise in completed research projects as part of a process to ensure overall greater ethical reflection and sensitivity to ethical issues in research.

## Data Availability

Not applicable.

## Abbreviations

DSMB: Data and safety monitoring board
IRB: Institutional review board
REC: Research ethics committee

## Footnotes

1 See our project website for more information: http://www.preaportal.org
